# The Effects of Childbirth Age on Maternal and Infant Outcomes in Pregnant Women

**Published:** 2018-06

**Authors:** Jing XU, Junhu WANG, Shuxia XUAN, Guiying FANG, Jinjing TIAN, Yucui TENG

**Affiliations:** 1. Dept. of Obstetrics, The Second People’s Hospital of Liaocheng, Liaocheng 252600, P.R. China; 2. Dept. of Obstetrics, The First Hospital of Hebei Medical University, Shijiazhuang 050000, P.R. China; 3. Dept. of Clinical Laboratory, The Second People’s Hospital of Liaocheng, Liaocheng 252600, P.R. China

**Keywords:** Childbirth, Age, Pregnancy complications, Adverse pregnancy outcomes

## Abstract

**Background:**

To investigate the effects of childbirth age on maternal and infant outcomes in pregnant women.

**Methods:**

The clinical data of 4552 singleton parturient women and their newborns treated in the Second People’s Hospital of Liaocheng, China from June 2015 to June 2017 were retrospectively analyzed. They were divided into group A (<20 yr old), group B (20–<30 yr old,), group C (30–<35 yr old), group D (35–<40 yr old), group E (≥40 yr old) according to the age of the parturient women. The incidence rates of pregnancy complications and adverse pregnancy outcomes of the pregnant and parturient women and their newborns in each group were compared.

**Results:**

With the increase of childbirth age, the incidence rates of pregnancy complications in pregnant women were increased gradually (*P*=0.028, 0.038, 0.042, 0.025, 0.012). The incidence rates of adverse pregnancy outcomes were increased gradually with the increase of childbirth age (*P*=0.006, 0.026, 0.010, 0.028). After correction of factors including pre-pregnancy body mass index (BMI), parity, gravidity and educational level, the incidence rate of cesarean section was reduced and the incidence rate of premature birth was increased in group A compared with those in group B. The incidence rates of cesarean section, premature birth, postpartum hemorrhage of pregnant women and the transference of newborns into NICU in group C, D and E were higher than those in group B (*P*=0.002, 0.019, 0.043, 0.015).

**Conclusion:**

Both low and high age pregnancy can increase the incidence rate of adverse pregnancy outcomes.

## Introduction

Elder pregnant and parturient women refer to the parturient women aged 35 yr or above at the expected date of childbirth. The childbirth age has now become a global problem, the fertility rate of Chinese pregnant and parturient women aged between 25–29 yr old has reduced to about 96%, while the fertility rate has increased to about 17% in women at the age of 35–39 and increased to about 5.74% in women at the age of 40–44 (1). With the comprehensive release of the “two-child” policy in recent years, the average childbirth age of women in China has also gradually increased. The fertility and the body condition related to childbearing of elderly pregnant women are low, and under the interaction of various factors, such as environmental pollution and psychological pressure, the incidence rate of pregnancy complications, such as hypertension of pregnancy and gestational diabetes, increases gradually (2). Furthermore, with the continuous integration of cultures of various countries, the incidence rate of low age pregnancy has become relatively high in recent years. Low age pregnant women often have diseases such as malnutrition and anemia, thus resulting in the high incidence rate of adverse pregnancy outcomes (3).

Therefore, to explore the influence of the childbirth age on maternal and infant outcomes in pregnant women has a great significance in carrying out the perinatal work and preventing the adverse pregnancy outcomes, analyzed in this study.

## Materials and Methods

### General materials

The clinical data of 4552 singleton parturient women and their newborns treated in the Second People’s Hospital of Liaocheng from June 2015 to June 2017 were retrospectively analyzed. Their age ranged from 18 to 45 yr old with an average of (28.91±3.48) yr old. The number of pregnancy was 1–6 times with an average of (1.86 + 0.79) times. The number of birth was 0–4 times with an average of (0.51 + 0.44) times.

This study was approved by the Ethics Committee of the Second People’s Hospital of Liaocheng. Signed written informed consents were obtained from the patients.

Inclusion criteria: 1) Singleton pregnancy and parturient women who had a normal delivery in our hospital, 2) pregnant and parturient women aged 18 yr old or above, and 3) pregnant and parturient women with complete clinical data and follow-up data.

Exclusion criteria: 1) Pregnant and parturient women complicated with a variety of acute or chronic diseases, 2) pregnant and parturient women complicated with a serious immune system disease, or 3) pregnant and parturient women complicated with a serious mental sickness.

### Methods

All pregnant and parturient women were followed up from the first antenatal examination in our hospital, and the incidence rates of pregnancy complications and adverse pregnancy outcomes of pregnant women and their newborns in each group were recorded and compared. 1) Pregnancy complications include intrahepatic cholestasis of pregnancy, pre-eclampsia, hypertensive disorder complicating pregnancy, adherent placenta, premature rupture of fetal membranes, postpartum hemorrhage, placenta praevia, abnormal amniotic fluid, gestational diabetes, placental abruption and anaemia, 2) adverse pregnancy outcomes include asphyxia neonatorum, low birth weight infants (LBWI), the transference of newborns into neonatal intensive care unit (NICU), neonatal birth defects, stillbirth, giant baby, postpartum hemorrhage, premature birth and cesarean section, 3) the clinical data of the pregnant and parturient women and their newborns were retrospectively analyzed and Logistic multifactor regression analysis was performed to analyze the relationships between the childbirth age and the occurrence risk of adverse pregnancy outcomes.

### Statistical methods

Statistical analysis was conducted by using Statistical Product and Service Solutions (SPSS) 20.0 (Chicago, IL, USA), and *x2* test was used for the enumeration data. After the correction of confounding factors, statistically significant adverse pregnancy outcomes were subjected to Logistic multifactor regression analysis, and *P*<0.05 suggested that the difference was statistically significant.

## Results

### Comparisons of pregnancy complications

With the increase of childbirth age, the incidence rates of intrahepatic cholestasis of pregnancy, preeclampsia, hypertensive disorder complicating pregnancy, adherent placenta, premature rupture of fetal membranes and gestational diabetes in pregnant women were increased gradually, and the differences were statistically significant (*P*<0.05) (Table 1).

**Table 1:** Comparisons of pregnancy complications [n (%)]

***Pregnancy complication***	***Group A (n=62)***	***Group B (n=2806)***	***Group C (n=1327)***	***Group D (n=309)***	***Group E (n=48)***	***x2***	***P***
Intrahepatic cholestasis of pregnancy	1(1.61)	48(1.71)	36(2.71)	11(3.55)	3(6.25)	6.276	0.028
Preeclampsia	1(1.61)	62(2.21)	385(29.01)	102(33.00)	3(6.25)	5.237	0.038
Hypertensive disorder complicating pregnancy	2(3.22)	101(3.59)	51(3.84)	31(10.03)	6(12.50)	4.739	0.042
Adherent placenta	2(3.22)	132(4.70)	68(5.12)	24(7.76)	5(10.41)	8.158	0.010
Premature rupture of fetal membranes	3(4.83)	163(5.80)	82(6.17)	38(12.29)	8(16.66)	6.536	0.025
Postpartum hemorrhage	3(4.83)	160(5.70)	76(5.72)	17(5.50)	3(6.25)	3.021	0.058
Placenta praevia	3(4.83)	115(4.09)	55(4.14)	16(5.17)	2(4.16)	1.854	0.071
Abnormal amniotic fluid	4(6.45)	179(6.37)	90(6.78)	18(5.82)	3(6.25)	2.539	0.063
Gestational diabetes	4(6.45)	275(9.80)	156(11.75)	56(18.12)	11(22.91)	7.921	0.012
Placental abruption	6(9.67)	28(0.99)	13(0.97)	1(0.32)	1(2.08)	1.594	0.085
Anemia	6(9.67)	283(10.08)	137(10.32)	30(9.70)	5(10.41)	1.438	0.089

### Comparisons of adverse pregnancy outcomes

The incidence rates of the transference of newborns into NICU, premature birth, postpartum hemorrhage and cesarean section were increased gradually with the increase of childbirth age, and the differences were statistically significant (*P* =0.006, 0.026, 0.010, 0.028). There was no statistically significant difference in the incidence rate of premature birth of newborns in group A compared with that in group E (*x2*=1.594, *P* =0.085) (Table 2).

**Table 2:** Comparisons of adverse pregnancy outcomes [n (%)]

***Adverse pregnancy outcome***	***Group A (n=62)***	***Group B (n=2806)***	***Group C (n=1327)***	***Group D (n=309)***	***Group E (n=48)***	***x2***	***P***
Asphyxia neonatorum	1(1.61)	54(1.92)	24(1.80)	6(1.94)	1(2.08)	1.542	0.087
LBWI	2(3.22)	81(2.88)	39(2.93)	9(2.91)	1(2.08)	2.285	0.065
Transference of newborns into NICU	3(4.83)	166(5.91)	99(7.46)	27(8.73)	6(12.50)	8.521	0.006
Neonatal birth defects	3(4.83)	15(0.53)	11(0.82)	3(0.97)	0(0.00)	1.329	0.091
Stillbirth	3(4.83)	134(4.77)	59(4.44)	15(4.85)	2(4.16)	1.689	0.080
Giant baby	4(6.45)	222(7.91)	101(7.61)	25(8.09)	5(10.41)	1.605	0.084
Postpartum hemorrhage	4(6.45)	150(5.34)	96(7.23)	23(7.44)	3(6.25)	6.536	0.026
Premature birth	6(9.67)	123(4.38)	77(5.80)	27(8.73)	6(12.50)	8.158	0.010
Cesarean section	14(22.58)	1063(37.88)	618(46.57)	205(66.34)	36(75.00)	6.276	0.028

### Occurrence risks of adverse pregnancy outcomes

After correction of factors including pre-pregnancy BMI, parity, gravidity and educational level, the incidence rate of cesarean section was reduced and the incidence rate of premature birth was increased in group A compared with those in group B. The incidence rates of cesarean section, premature birth, postpartum hemorrhage and the transference of newborns into NICU in group C, D and E were higher than those in group B, and the differences were statistically significant (*P* =0.002, 0.019, 0.043, 0.015) (Table 3).

**Table 3:** Occurrence risks of adverse pregnancy outcomes

***Group***	***Case (n)***	***Premature birth***		***Postpartum hemorrhage***		***Cesarean section***		***Transference of newborns into NICU***	
		***OR***	**95% *confidence interval* (*CI*)**	***OR***	**95% *CI***	**OR**	**95% *CI***	***OR***	**95% *CI***
Group A	62	2.144	1.256–3.662	1.181	0.647–2.156	0.549	0.391–0.771	0.990	0.513–1.913
Group B	2806	1.000	-	1.000	-	1.000	-	1.000	-
Group C	1327	1.219	1.021–1.455	1.224	1.048–1.429	1.373	1.268–1.486	1.236	1.059–1.443
Group D	309	1.644	1.251–2.161	1.225	0.959–1.642	2.960	2.550–3.435	1.337	1.018–1.756
Group E	48	2.141	1.209–3.792	1.218	0.631–2.352	4.332	2.920–6.426	2.389	1.385–4.121

## Discussion

With the rapid development of China’s economy, most childbearing-age women choose to postpone their childbirth age. Besides, with the continuous application of assisted reproductive technology, the pregnancy rate of some primary or secondary infertile patients has increased, which also increases the average maternal childbirth age. The elderly women are characterized by ovarian dysfunction and diminished fertility, and some of them are combined with chronic diseases such as hypertension and diabetes, which lead to a higher incidence rate of adverse pregnancy outcomes (4,5). At the same time, as a result of the continuous development of society and the integration of the cultures of various countries, the number of pregnant and parturient women has been increasing in recent years. Due to the fact that all the functions of young pregnant women have not yet reached the standard of pregnancy, and they often suffer from anemia and malnutrition, it is hard for them to provide appropriate conditions for the development of their fetuses, so the incidence rate of adverse pregnancy outcomes is also high (6). Therefore, this study explored the relationships between childbirth age and maternal and infant outcomes, so as to improve the pregnancy outcomes by taking the targeted perinatal care as the basis.

The results of this study showed that the incidence rates of intrahepatic cholestasis of pregnancy, preeclampsia, hypertensive disorder complicating pregnancy, adherent placenta, premature rupture of fetal membranes and gestational diabetes in pregnant women were increased gradually with the increase of childbirth age, and the differences were statistically significant (*P* <0.05). The incidence rates of the transference of newborns into NICU, premature birth, postpartum hemorrhage and cesarean section were increased gradually with the increase of childbirth age, and the differences were statistically significant (*P*<0.05). There was no statistically significant difference in the incidence rate of premature birth of newborns in group A compared with that in group E (*P*>0.05). For young pregnant and parturient women, their own growth and development have not been completed, and the fertility is relatively low. The fetus needs a lot of nutrient supplies during the pregnancy, so the young pregnant women are difficult to meet the needs of their own and their fetuses during the pregnancy. Therefore, the fetal growth and development are limited and even the incidence rate of premature birth is relatively high (7, 8). Therefore, we should try to improve the nutritional status of the young pregnant and parturient women clinically so as to improve the pregnancy outcomes. It is also necessary to increase the knowledge through propaganda and education to avoid low age women from pregnancy. For elderly women, the low islet function and insulin sensitivity, the impaired function of islet beta cells and the excessive secretion of glucocorticoid, such as placental prolactin, are easy to induce insulin resistance. So, the incidence rate of gestational diabetes of elderly pregnant women is high, and the pregnant and parturient women with gestational diabetes can be complicated with infection, premature birth and abortion, and even can cause ketoacidosis in severe cases, endangering their lives (9). At the same time, there are possibilities of giant baby, excessive amniotic fluid, asphyxia, and even stillbirth in severe occasions. Therefore, it is necessary to increase the screening of blood glucose of elderly pregnant women clinically in order to detect patients with gestational diabetes as soon as possible, control blood sugar and reduce the incidence rate of adverse pregnancy outcomes (10). Besides, elderly women tend to have chromosomal abnormality of oocyte, which leads to a high incidence rate of fetal chromosomal abnormality, ultimately increasing the incidence rates of abortion and premature birth (11). Moreover, with the increase of age of females, the progressive injury of vascular endothelial cells in myometrium, the replacement of smooth muscle fibers by collagen fibers, the reduced ability of vasoconstriction and arteriosclerosis can cause oxidative stress reaction or local ischemia of local placenta, peripheral vasospasm, decrease the cardiac systolic stroke volume and weaken the heart receptivity, thus leading to utero-placental ischemia, fetal growth restriction, gestational hypertension of the pregnant women and increase the premature birth rate of the fetus(12,13).

We also found in clinic that the pelvic joints of the elderly pregnant and parturient women are hard, which are difficult to dilate, and the contraction ability of the uterine myometrium is poor, so it may take a long delivery time if natural childbirth is carried out, and some patients even suffer from dystocia. Therefore, cesarean section is more often adopted in elderly pregnant and parturient women clinically (14).

## Conclusion

Both low and high age pregnancy can increase the incidence rate of adverse pregnancy outcomes, the health care work during pregnancy should be taken seriously clinically and related pregnancy complications should be treated actively so as to ensure the maternal and neonatal health.

## Ethical considerations

Ethical issues (Including plagiarism, informed consent, misconduct, data fabrication and/or falsification, double publication and/or submission, redundancy, etc.) have been completely observed by the authors.
